# Thiolate-Capped CdSe/ZnS Core-Shell Quantum Dots for the Sensitive Detection of Glucose

**DOI:** 10.3390/s17071537

**Published:** 2017-07-01

**Authors:** Samsulida Abd. Rahman, Nurhayati Ariffin, Nor Azah Yusof, Jaafar Abdullah, Faruq Mohammad, Zuhana Ahmad Zubir, Nik Mohd Azmi Nik Abd. Aziz

**Affiliations:** 1 Industrial Biotechnology Research Centre (IBRC), SIRIM Berhad, No. 1, Persiaran Dato’ Menteri, Section 2, P.O. Box 7035, 40700 Shah Alam, Selangor, Malaysia; sulida@sirim.my (S.A.R.); ahayati@sirim.my (N.A.); 2 Advanced Materials and Nanotechnology Laboratory, Institute of Advanced Technology, Universiti Putra Malaysia, 43400 UPM Serdang, Malaysia; jafar@upm.edu.my; 3 Department of Chemistry, Faculty of Science, Universiti Putra Malaysia, 43400 UPM, Serdang, Malaysia; 4 Surfactant Research Chair, Department of Chemistry, College of Science, King Saud University, P.O. Box 2455, Riyadh 11451, Saudi Arabia; 5 Advance Material Research Centre (AMREC), SIRIM Berhad, Lot 34, Jalan Hi-Tech 2/3, Kulim Hi-Tech Park, 09000 Kulim, Malaysia; zuhana@sirim.my (Z.A.Z.); nikazmi@sirim.my (N.M.A.N.A.A.)

**Keywords:** Cdse-ZnS, core-shell quantum dots, semiconducting, glucose sensing, surface modification

## Abstract

A semiconducting water-soluble core-shell quantum dots (QDs) system capped with thiolated ligand was used in this study for the sensitive detection of glucose in aqueous samples. The QDs selected are of CdSe-coated ZnS and were prepared in house based on a hot injection technique. The formation of ZnS shell at the outer surface of CdSe core was made via a specific process namely, SILAR (successive ionic layer adsorption and reaction). The distribution, morphology, and optical characteristics of the prepared core-shell QDs were assessed by transmission electron microscopy (TEM) and spectrofluorescence, respectively. From the analysis, the results show that the mean particle size of prepared QDs is in the range of 10–12 nm and that the optimum emission condition was displayed at 620 nm. Further, the prepared CdSe/ZnS core shell QDs were modified by means of a room temperature ligand-exchange method that involves six organic ligands, *L*-cysteine, *L*-histidine, thio-glycolic acid (TGA or mercapto-acetic acid, MAA), mercapto-propionic acid (MPA), mercapto-succinic acid (MSA), and mercapto-undecanoic acid (MUA). This process was chosen in order to maintain a very dense water solubilizing environment around the QDs surface. From the analysis, the results show that the CdSe/ZnS capped with TGA (CdSe/ZnS-TGA) exhibited the strongest fluorescence emission as compared to others; hence, it was tested further for the glucose detection after their treatment with glucose oxidase (GOx) and horseradish peroxidase (HRP) enzymes. Here in this study, the glucose detection is based on the fluorescence quenching effect of the QDs, which is correlated to the oxidative reactions occurred between the conjugated enzymes and glucose. From the analysis of results, it can be inferred that the resultant GOx:HRP/CdSe/ZnS-TGA QDs system can be a suitable platform for the fluorescence-based determination of glucose in the real samples.

## 1. Introduction

In general, the nanomaterials maintain the special characteristics, which include the substantial surface area, superior reaction surface activity, and higher catalytic efficiency, to mention some [1]. For these reasons, the nanomaterials are considered to be the prospective transducers in enzyme-based bioconjugated sensor-related purposes. The substantial surface area of nanomaterials allows for the efficient adsorption of enzymes to the solid surfaces, in addition to minimizing the enzyme aggregation and protein unfolding, thereby supporting the formation of a more stable enzyme-loaded nanoparticulate system [2,3]. For the majority of biosensor-related applications, the previously reported nanomaterials include gold [4], carbon nanotubes [5], magnetic iron oxide [6], titania [7], silica [8], and quantum dots (QDs) [9,10,11,12,13,14]. Among all these nanomaterials, QDs have been found to be more favorable for sensor applications as they exhibit broad excitation and narrow emission wavelengths, in addition to allowing their emission wavelengths to be fine-tuned. Additionally, the other attractive features of QDs, i.e., their extremely luminescent and photoresistant properties (due to high surface-to-volume ratio, catalytic efficiency, and reaction activity surface), might be particularly useful in the biosensors sector [1]. The water-soluble QDs have given rise to an increasing passion towards the biosensors and bioimaging because of their biocompatible characteristics in the physiological medium. In addition, the utilization of QDs for the development of enzyme-conjugated systems help in two different ways, i.e., (1) by providing a strong solid support for the immobilization of the enzymes and (2) by acting as a fluorescence sensing probe while taking advantage of the changes in fluorescence intensity [15]. The fluorescence emission wavelengths of QDs are mostly influenced by the changes in particle sizes and surface covered ligands as the charges are strongly inclined by the nature of ligands and all of which finally responsible for the biomolecule interactions [16].

In healthcare and biomedical sector, most of the scientific work reported to date deals with the usage of QDs for diagnostic and biosensory systems such as cancer cell labelling [17], diseased cell imaging [18], drug delivery, and virus detection [19], to mention some. Apart from that, QDs are also the most familiar agents for the sensory detection of glucose levels, and one most popular QD material in that aspect is the CdSe/ZnS core-shell QDs. It was reported that the CdSe/ZnS core-shell QDs are extremely high fluorescence intensity agents in the visible spectrum and that their enhancement in the chemical and photostability is mainly due to the ZnS outer layer [20,21,22]. The common method for the synthesis of CdSe/ZnS core-shell QDs involves a high temperature organic solvent method, where the particles formed are stabilized in hydrophobic solvents like trioctyl phosphine oxide (TOPO) and oleic acid (OA). Since the QDs formed by this approach result in the generation of hydrophobic particles and in order to avoid this, several methods have been studied to substitute TOPO and/or OA groups with other organic ligands with a hydrophilic nature. However, the exchange of TOPO and/or OA with other organic ligands has been found to generate problems such as a lower quantum yield, a loss of fluorescence intensity, larger-sized particles, and a compromising of biocompatibility [23,24,25,26]. Gill et al. (2005) previously reported that the quantum yield of their synthesized QDs was reduced to 50% as the CdSe/ZnS QDs were modified with mercapto-propionic acid (MPA) [24]. In a similar way, Stsiapura et al. (2006) found from their studies that the quantum yield of their synthesized QDs depreciated twofold when mixtures of mercapto-succinic acid (MSA) and thioglycolic acid (TGA; or mercapto-acetic acid (MAA)) were used as ligand exchangers [25]. In addition to quantum yield loss, the QDs were also reported to exhibit less colloidal stability, as they have a greater tendency to form aggregates rather than residing individually in the dispersing medium.

Based on the facts about the synthesis and stability of QDs in the colloidal medium, the present work is aimed to prepare the CdSe/ZnS core-shell QDs with water-soluble behavior and can be developed to serve as a glucose biosensor. For that, we first prepared the CdSe/ZnS core-shell QDs using a high temperature organic solvent method that uses hydrophobic TOPO and OA as capping agents in order to stabilize the particles as soon as they are formed [26]. Further, to enhance their dispersibility in aqueous solutions, the ligand exchange method was applied to modify the surface of QDs, in addition to fine-tuning the surface to match for biosensing applications. For that, the heterobifunctional ligands such as the mercapto-carbonic acid and thiolated ligands were used to replace the TOPO/OA, where the mercapto or thiolated end is bound to the surface of QDs so that the carboxyl moiety remains free and can offer enough water solubility [19,27]. In general, the presence of high molecular weight compounds on the surface restricts the usefulness of the core-shell QDs towards biosensing applications, and this is due to the blockage of electron transfer reaching the core QDs. In our case, the coating of QDs with mercapto-carbonic or thiolated ligands helps to prevent such limitations of electron movement, thereby serving as the best water solubilizing shell to the CdSe/ZnS QDs. Thus, the formed composite was thoroughly characterized by means of transmission electron microscopy (TEM), fluorescence measurements, and further tested to see their effects for the detection of glucose following the QDs loading with that of glucose oxidase enzyme (GOx) and horseradish peroxidase (HRP) enzymes.

## 2. Materials and Methods

### 2.1. Chemicals

*N*-Ethyl-3-(3-dimethylaminopropyl) carbodiimide (EDC), N-hydroxy sulfosuccinimide (sulfo-NHS), glucose oxidase (GOx), and horseradish peroxidase (HRP) were purchased from Sigma (Selangor, Malaysia). The solution of PBS buffer, pH 7, were prepared in house by the addition of disodium hydrogen diphosphate (Na_2_HPO_4_) and sodium dihydrogen phosphate (NaH_2_PO_4_) until it reaches pH 7. Both Na_2_HPO_4_ and NaH_2_PO_4_ were purchased from Scharlau (Selangor, Malaysia). All the starting materials for enzyme conjugation and glucose detection ordered were of highest grade and were used directly without any further purification.

### 2.2. Synthesis of CdSe/ZnS-TGA QDs

For the synthesis, we first started with the preparation of 0.1 M Cd and Se precursor solutions separately; and for that about 150 mg of Se powder was dissolved in 20 mL of tri-*n*-octylphosphine (TOP), stirred at 200 °C temperature in nitrogen atmosphere until the solution becomes clear. Similarly, the Cd precursor was prepared by mixing 160 mg of cadmium oxide (CdO), 3.5 mL of OA and 10 mL of 1-octadecene (ODE) for 30 min at 150 °C and further heated up to 280 °C in nitrogen atmosphere. When we see the solution becoming clear, the temperature was reduced to 225 °C and then added about 5 mL of Se precursor solution to the hot flask, stirred for 10 min and stopped the heating process. Now we added about 15 mL of methanol to the reaction mixture so as to stop the size growth and then washing with acetone solution was continued in order to remove any organics, and the washing process was repeated until the solution becomes opaque. The solid precipitate obtained after the centrifugation at 5000 rpm for 15 min was collected by discarding the supernatant, dried in vacuum before using for the shelling process. Similarly, 0.1 M of zinc precursor solution was prepared by adding 0.13 g of ZnO to the mixture of OA and ODE (1.5 mL and 13.5 mL respectively) at 200 °C. Additionally, the sulfur precursor solution (0.1 M) was prepared by dissolving sulfur (0.02 g) in ODE (15 mL) at 200 °C. All the precursor solutions were maintained under nitrogen atmosphere until further use.

For obtaining the CdSe/ZnS (core/shell) QDs, about 0.1 g of CdSe (dissolved in hexane) was added to a solution mixture containing 0.6 mL of ODE and 0.2 mL of OA in a 25 mL reaction vessel, heated up to 100 °C for 30 min. Following the period, nitrogen flow was maintained into the reaction vessel for another 1 h at 100 °C so as to remove the hexane and any other undesired materials of low vapor pressure, and then the solution was heated to 160 °C to allow for shell growth. The amounts of solutions injected into the reaction mixture at each step were as follows: We started with 0.2 mL of Cd and S solutions for the first layer and the temperature was slowly raised to ~180 °C for 5 min. For the second layer, 0.4 mL of each solution and the temperature was slowly raised to ~200 °C for 5 min, and similarly 0.6 mL of each solution for the third layer at ~220 °C temperature for ~5 min, 0.8 mL of each solution for the fourth layer at ~240 °C for 5 min, and 1 mL of each for the fifth layer at 260 °C for 30 min. For each injection, small aliquots were extracted from the mixture after 5 min and mixed with toluene to record the absorption spectrum. Methanol was added to stop the growth, and the mixture was purified using the same method as the core.

In order to convert CdSe/ZnS QDs containing OA ligand (organic soluble) into aqueous dispersible, we first dissolved the CdSe/ZnS powder in toluene, which was then supplemented with ethanol in a 1:1 ratio (i.e., 1 mL of CdSe/ZnS in toluene with 1 mL of ethanol) followed by shaking. After that, the solution mixture was centrifuged at 3000 rpm for 15 min, the supernatant was discarded, and the precipitate was washed with ethanol, and the centrifugation process was repeated 2–3 times. After the required centrifugation, the precipitated product was collected and re-dissolved in toluene; to this, a QD solution, an excess amount of thiol-terminated ligands (*L*-cysteine, *L*-Histidine, TGA, MPA, MSA and MUA), was added and sonicated for 30 min. After the sonication, the mixture was kept at room temperature for one day and then centrifuged at 5000 rpm for 5 min and the obtained precipitate was dried in a vacuum desiccator for 1 h. Finally, the CdSe/ZnS-capped thiolated ligands were dissolved in the PBS pH 7, and the mixture was then centrifuged at 5000 rpm for 5 min, the supernatant was removed, and the collected precipitate was dried in a vacuum desiccator for 1 h. Thus, the obtained CdSe/ZnS capped with respective ligands with an aqueous dispersible nature was re-dissolved in PBS pH 7 until further use.

### 2.3. Conjugation of CdSe/ZnS-TGA QDs with GOx:HRP Enzymes in an Aqueous System

For the conjugation of enzymes to the QDs, we first added 2.3 µL of EDC and 1 mg of NHS to a beaker containing 5 mL of CdSe/ZnS-TGA. Both solutions were mixed together with a magnetic stirrer for 30 min and then supplemented with 25 µL of GOx and 10 µL of HRP. The stirring was continued for another 2 h before the centrifugation process at 12,000 rpm for 10 min. Following the centrifugation, the supernatant was separated out and the precipitate was re-dissolved in PBS pH 7 and stored at 4 °C for the next usage. All characterizations of the GOx:HRP-conjugated CdSe/ZnS-TGA QDs were performed using the spectrofluorescence technique. The characterization of enzyme conjugation and interaction were also evaluated using different mixing ratios of QDs and enzyme (1:1, 1:2, 1:3, 1:4) in the assay system. Further, the analytical performance of the bioconjugated QDs-enzyme system was evaluated by analyzing the stability, interference, repeatability, and reproducibility.

### 2.4. Instrumental Analysis

The Ultraviolet–Visible (UV-Vis) absorption spectroscopic measurements were performed on Perkin Elmer LAMBDA 25 UV/VIS system and the samples were prepared by dissolving the CdSe/ZnS particles in an aqueous solution and were run in the wavelength range of 300–800 nm with an absorbance less than 0.1 at a 480 nm wavelength. For the photoluminescence study, the fluorescence spectrums were recorded using the Ocean Optics QE65000 and Flouromax-4 (Horiba Jobin Yvon) spectrophotometers in the wavelength range of 500–700 nm. The samples for the spectrofluorescence study were placed in a quartz cuvette, and the emission spectrum of each sample was measured using the EFOS Novacure spectrophotometer with a mercury light source; all the samples were excited at a wavelength of 375 nm. For the transmission electron microscopy (TEM) analysis, a Philips Technai20 instrument connected with an EDAX analyzer was used (operating at 200 KV, 100X to 1000KX range). The samples for TEM analysis were prepared by placing tiny drops of QD suspension in water on a carbon-coated 400 mesh copper grid.

## 3. Results and Discussion

**Figure 1:**
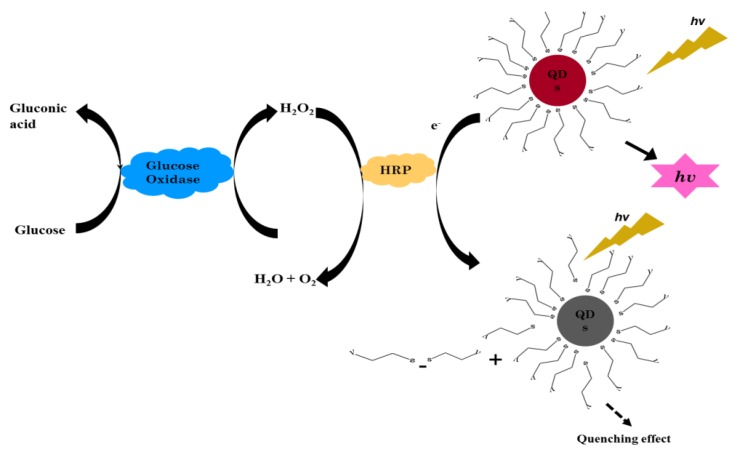
Schematic representation of successive reactions involved in the sensitive detection of glucose by means of GO_X_:HRP/CdSe/ZnS-TGA QD system.

The basic principle for the detection of glucose by involving the CdSe/ZnS-capped TGA nanomaterials is shown in Figure 1. Here, the determination of glucose in this research depends on the enzymatic reaction of glucose and the effect of presence of H_2_O_2_ on the intensity of QDs fluorescence. In the presence of glucose, GOx loaded onto the substrate catalyzes the available glucose into gluconic acid by means of an oxidation process using the oxygen molecule as an electron acceptor and in turn produces H_2_O_2_ simultaneously. The exchange of the electron happens at the outer surface of the core-shell QDs, whereby H_2_O_2_ is reduced to oxygen and H_2_O, which traps the electron holes at the surface of the QDs. This further results in the generation of non-fluorescent QDs and in some instances causes a reduction in the fluorescence intensity, i.e., the higher glucose concentrations used, the more H_2_O_2_ produced and thus the greater the quenching effect.

**Figure 2:**
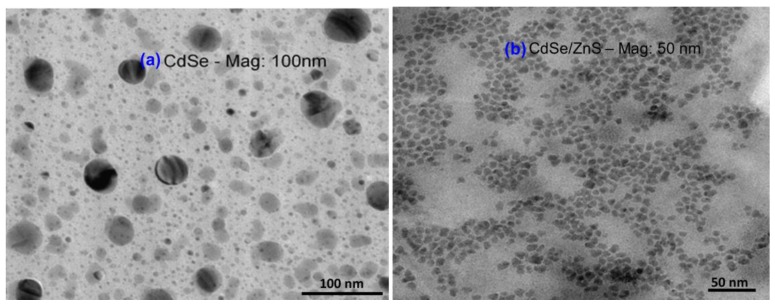
TEM results of (**a**) CdSe core, and (**b**) CdSe/ZnS core-shell QDs with the magnifications of 100 nm and 50 nm, respectively.

TEM images of both CdSe core and CdSe/ZnS core-shell QDs are shown in the Figure 2a,b and from these figures, it can be seen that the sizes of the CdSe and CdSe/ZnS particles are in the range of 3–3.2 nm and 10–12 nm, respectively. It can also be seen from the images that the particles are uniform and monodispersed, thereby providing evidence for the successive coating of the QDs with that of ligands.

**Figure 3:**
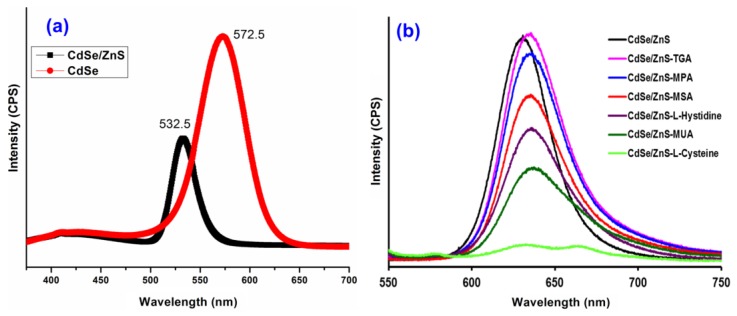
(**a**) Fluorescence intensity peaks for CdSe and CdSe/ZnS QDs when recorded using PL measurements and (**b**) fluorescence for the CdSe/ZnS QDs with different surface ligands (TGA, MPA, MSA, *L*-Histidine, MUA, and *L*-Cysteine) using spectrofluorescence measurement.

For the photoluminescence (PL) measurements in general, the samples are usually dissolved in a non-polar solvent, and for this reason, we have chosen hexane solvent as the dispersing medium for our synthesized QDs. The PL spectrum of the QDs sample before and after its coating with the ZnS shell is shown in Figure 3a. It can be seen from Figure 3a that the PL peak for the CdSe core was observed to be around 532.5 nm, and the full width at half maximum (FWHM) was 28.5 nm, thereby indicating for the formation of the monodispersed nanocrystals. However, the PL peak for the CdSe/Zns core-shell QDs was observed at 572.5 nm, and the PL intensity observed to be quite higher for the encapsulation. Further, the wavelength shifted to about 40 nm between the core and the core/shell type, which may be due to the reheating of core before encapsulating that cause an increase in the diameter of core.

Further, in order to make the particles water-dispersible, the surface of CdSe/ZnS core-shell QDs (OA organic capping) was replaced with six different hydrophilic ligands (TGA, MPA, MSA, *L*-Histidine, MUA, and *L*-Cysteine). Figure 3b shows the fluorescence intensities of the capped CdSe/ZnS-OA (without modification) as well as those of CdSe/ZnS-TGA, CdSe/ZnS-MPA, CdSe/ZnS-MSA, CdSe/ZnS-*L*-histidine, CdSe/ZnS-MUA, and CdSe/ZnS-*L*-cysteine, respectively. The fluorescent peaks in Figure 3a,b differ in absorption maxima at 572 nm and 620 nm, respectively. This difference in fluorescent peaks is due to the variation in the equipment used and their principles, i.e. we used PL measurements for the fluorescence intensity measurements between CdSe (core) and CdSe/ZnS (core-shell) (Figure 3a), while a spectrofluorometer was employed for the comparison of fluorescence intensity of CdSe/ZnS coated with different ligands. It is clearly indicated here that the fluorescent intensity of CdSe/ZnS capped with *L*-histidine, MUA, and *L*-cysteine seems to decrease compared to pure CdSe/ZnS, and the observation of such results is mostly due to the formation of agglomerated structures and insolubility when they are re-dissolved in PBS buffer, pH 7. However, the CdSe/ZnS capped with TGA, MPA, and MSA showed no signs of agglomeration and are fully dissolved in PBS buffer, pH 7, thereby indicating for their high stability in aqueous dispersions. In general, the fluorescence intensity mostly affected by the presence of surface ligands, i.e. the ligand with the longest chain or complex structure strongly reduces intensity, as compared to the one with simple chain or non-bulky group molecules. Here in our case, TGA maintains only two carbons in its chain and holds a simple structure, which was followed by MPA (three carbon chain), MSA (four carbons), *L*-histidine (four carbons and a substituent having ring structure), MUA (11 carbons), and finally *L*-cystein (3 carbons with spatial arrangement of groups) [28]. Hence, based on the surface ligand and its type, the observed order of fluorescence intensities for the CdSe/ZnS QDs with different ligands can be explained.

**Figure 4:**
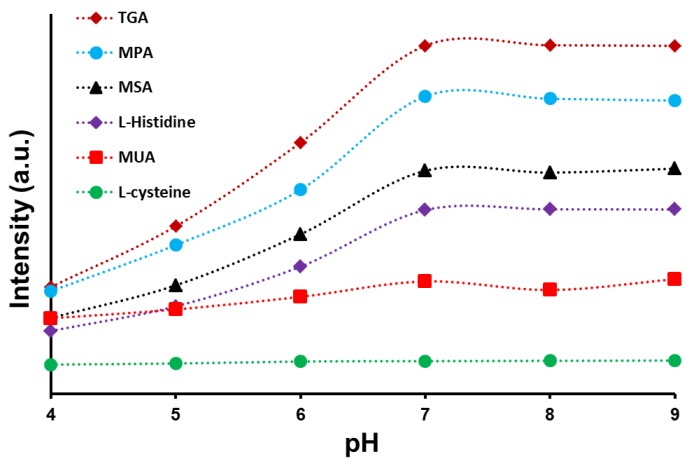
Fluorescence intensity for CdSe/ZnS QDs coated with different surface ligands of TGA, MUA, MPA, MSA, *L*-cystein, and L-histidine under different solution pH values.

The fluorescent intensity of CdSe/ZnS capped with different ligands under different pH conditions can further provide evidence for the functional group changes in accordance with the solution pH. Figure 4 shows the effects of pH buffer ranging from 4–9 on the fluorescent intensity of CdSe/ZnS capped with different organic ligands (as mentioned previously). From the figure, the fluorescent intensity for CdSe/ZnS capped with TGA increased as the pH value increased from 4 to 7, and then became nearly constant when the pH value was 7 or greater. The fluorescent intensity increased 1.5 times for all three solutions when the solution pH increased from 4 to 5, from 5 to 6, and from 6 to 7, respectively. Similar to CdSe/ZnS capped with other ligands (MPA, MSA, *L*-histidine, MUA and *L*-cysteine), the fluorescent intensity increased as the solution pH value increased from 4 to 7, while the intensity became nearly constant when the pH value was greater than 7. Additionally, the intensity seems to be increased nearly 1.4 times for the CdSe/ZnS capped with MPA, MSA, and *L*-histidine, separately, and 1.1 times for the CdSe/ZnS capped with MUA and *L*-cysteine when the solution pH increased from 4 to 7. When the solution pH value was greater than 7, the mean fluorescent intensity is not affected, meaning that no changes to the surface groups are occurring.

We see from the results that among the three ligands (TGA, MPA, and MSA), the CdSe/ZnS-TGA sample produced the highest fluorescent intensity due to the smaller ligand size as compared to the other two samples (CdSe/ZnS-MPA and CdSe/ZnS-MSA). The chain size in these ligands follow the order of TGA < MPA < MSA, where the decrease in the aggregation levels by means of decreased steric repulsions (more stable particles in solution) can be expected and all of which contributes finally for an enhancement in the mean fluorescence intensity [2,28]. With the same size and ligand chain length principle, the observed highest fluorescence intensity of TGA (comparable to OA-capped CdSe/ZnS QDs) can be explained by relating it with that of MPA, MSA, *L*-histidine, MUA, and *L*-cysteine chain length. In addition, it can be mentioned here that the CdSe/ZnS-TGA QDs were observed to be the most efficient probes, as they are water-dispersible, biocompatible, and maintain a fluorescence behavior equal to that of the OA-capped CdSe/ZnS QDs. The biocompatible property is contributed by the OA and TGA groups, as we found from our earlier studies that any ligand which has the functional groups in its structure such as amine, thiol, or unsaturation was observed to maintain some inbuilt anti-oxidative properties. This property is strong enough to protect the cells from the toxic responses generated by the CdSe or ZnS QDs individually or in a combination form [29]. Furthermore, the ligand capping also provides the CdSe/ZnS QDs with a negative surface charge distribution that can provide direct self-assembly with other molecules with a positive charge. Theoretically, the CdSe/ZnS-TGA QDs can be extended for the detection of other analytes if appropriate conditions are established. From the analysis, therefore, we came to a conclusion that the CdSe/ZnS-TGA QDs can serve as the most efficient probes (as compared to MPA, MUA, MSA, *L*-histidine, and *L*-cysteine-coated QDs) due to its enhanced fluorescence, water solubility, and biocompatible properties, which can be further exploited for biomedical applications and, in the present case, for glucose sensing through enzyme loading.

**Figure 5:**
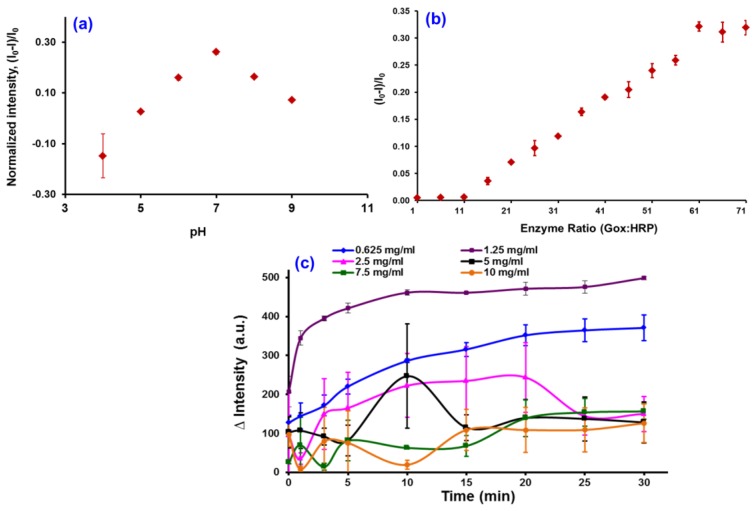
The effect of pH (**a**), enzyme ratio (**b**), and QD concentration (**c**) towards the fluorescence intensity.

The optimization parameters that were studied are of the effect of pH, enzyme ratio, and QD concentration, as these are very important for glucose biosensing and were analyzed using a mixture of GOx/HRP and TGA-QDs, and the results are displayed in Figure 5. Figure 5a shows the optimization of pH buffer on the fluorescence intensity quenching of the CdSe/ZnS-capped TGA. From the graph (Figure 5a), it can be seen that the quenching effect of the CdSe/ZnS-capped TGA increases with an increase in the pH buffer until it reaches an optimum condition of pH 7. This is due to the fact that, at pH 7, the glucose molecule, being in a cyclic hemiacetal form, may exist in the two isomeric forms *β*-*D*-glucopyranose (63.6%) and *α*-*D*-glucopyranose (36.4%). In association with our QDs probe, some specific binding occurs between the GOx and *β*-*D*-glucopyranose forms, while no such binding can occur with the *α*-*D*-glucopyranose form. The equilibrium state between the *α*-*β* glucopyranose forms seems to be pushed towards the *β*-side as the amount of consumption is more for the *β*-form as against the *α*-form of gluocopyranose. This equilibrium enabled GOx to oxidize all of the glucose in solution. Further, a drop in the intensity of the QDs after pH 7 may be due to the denaturation of the GOx enzyme in the basic environment.

The effect of normalized intensity towards the GOx:HRP ratio is shown in Figure 5b, and the graph shows that the optimum ratio of GOx:HRP is 3:2. We observed that the quenching effect of the QDs is greatest when the GOx:HRP enzyme ratio is maintained at 3:2, and the reflection of such an effect may be due to the stability of the system, so the same ratio was repeated for the following experiments. Further, the effect of the QDs concentration studied in the range of 0.625–10.0 mg/mL of CdSe/ZnS and is shown in Figure 5c. The results of the study (Figure 5c) indicate that the highest intensity was obtained for the 1.25 mg/mL concentration of the QDs. However, the intensity of the QDs was decreased when the highest QDs concentration was introduced into the reaction, and this may be due to the quenching effects of CdSe/ZnS at higher concentration, thereby resulting in the enzyme denaturation/degradation. Therefore, based on this study, a 1.25 mg/mL concentration of QDs, a 3:2 ratio of GOx:HRP enzymes ratio, and pH 7 were chosen as the optimal conditions for the subsequent reactions.

**Figure 6:**
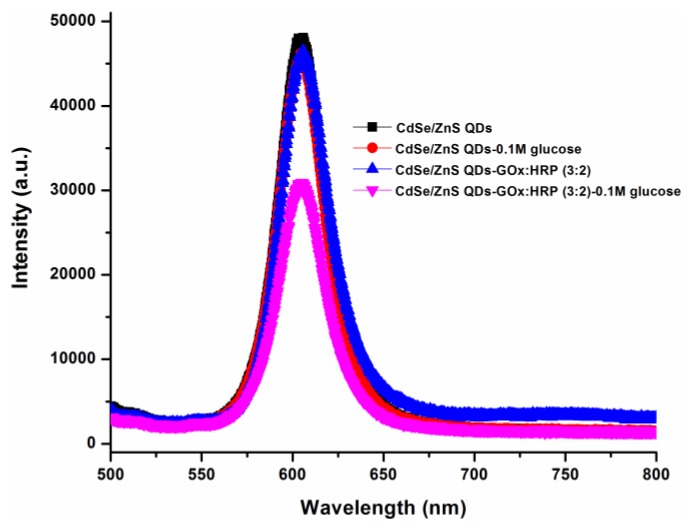
The fluorescence spectrums of CdSe/ZnS QDs along with different combinations of its loading with GOx:HRP enzyme and in the presence of 0.1 M glucose. All spectra were recorded after mixing the components for 30 min.

The reaction mechanism for glucose detection that depends on the effect of CdSe/ZnS fluorescence intensity quenching was shown to be successful, and the results are shown in Figure 6. From the figure, one can clearly see that the highest intensity was observed for the as-prepared QDs when they are maintained in the absence of GOx:HRP enzymes and glucose. However, in the presence of GOx:HRP and glucose, the reaction occurred and resulted in the highest quenching of QD intensity; thus, we observed the fluorescence intensity to be the lowest, as compared to all other combinations.

**Figure 7:**
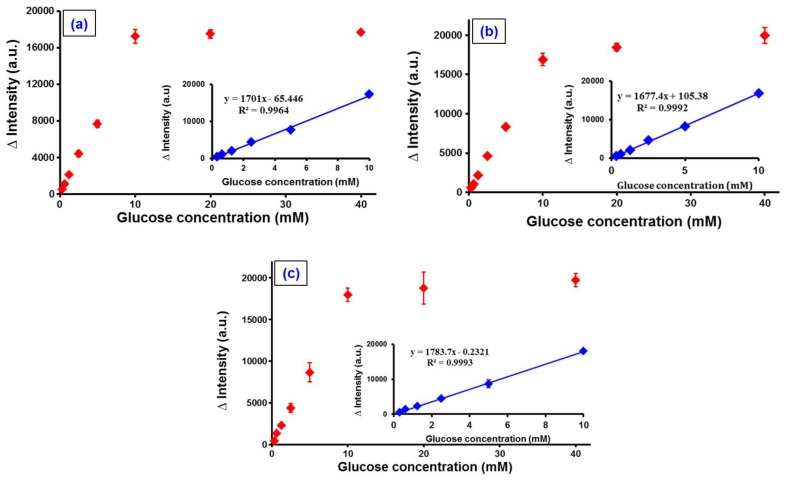
Fluorescence intensity of CdSe/ZnS-capped TGA core shell QDs via various glucose levels from 0 to 40 mM. The inset shows the relationship between intensity and glucose concentration. The calibration curves were produced in triplicate ((**a**–**c**) labels in the figure corresponds to the repetition thrice) using different batches of CdSe/ZnS.

Further, the calibration studies were performed several times using different concentrations of glucose varying from 0 to 40 mM (0, 0.03, 0.07, 0.15, 0.31, 0.62, 1.25, 2.5, 5, 10, 20, and 40 mM, respectively), and the assay was repeated thrice for each measurement where the results are displayed in Figure 7a–c. In addition, the inset of Figure 7 shows that there is a good linearity between the quenched intensity of the CdSe/ZnS QDs and the glucose levels when recorded in the range of 0–10 mM. From the results, we calculated the corresponding regression coefficient to be about 0.998, while the limit of detection (LOD) obtained was 0.045 mM. The LOD was calculated through 3*σ*/*s*, where *s* is the slope of calibration, while *σ* is the standard deviation of the corrected blank from the fluorescence signals of the CdSe/ZnS-TGA QDs. The LOD of this study was slightly lower than the other previously reported studies that involve QDs for the glucose detection (Table 1). Further, our proposed method provides a good reproducibility with the relative standard deviation (RSD) of 3.33% for a 10-fold repeated detection of 1.25 mM glucose. Finally, we believe that our method is simple, reliable, and can offer a practical approach for the non-invasive detection of glucose in the real-time samples.

## 4. Conclusions

In summary, we have demonstrated the CdSe/ZnS capped TGA water-soluble core-shell QDs for the qualitative and quantitative determination of glucose. The detection principle was based on the fact that the spectrofluorescence signals of the prepared CdSe/ZnS-capped TGA QDs can be quenched by the presence of glucose successively. The parameters for fluorescence quenching reaction includes the solution pH, enzyme ratio, and the QD concentration; further, the optimal conditions for the observation of efficient fluorescence towards glucose detection by the use of QDs have been very well discussed. Since the glucose detection principle is based on the fluorescence quenching effect and we observed that in the presence of 0.1 mM glucose, the fluorescence intensity of the bioconjugated QDs was quenched about 12,000 a.u. Further, the bioconjugated GOx:HRP/QDs-capped TGA was analyzed with known concentrations of glucose and indicated that the quenching of fluorescence intensity is proportionate to the glucose concentration. An extremely good linearity for the glucose determination in the range of 0–10 mM was observed, in addition to obtaining the LOD to be 0.045 mM. Thus, from the analysis of the results in this study, it can be concluded that our synthesized QDs are accurate, sensitive, and can be applied as the fluorescence nanosensor for the detection of glucose in real samples.

## Figures and Tables

**Table 1:** Detection limits and ranges for optically based glucose detection using QDs.

QDs	Ligands	Enzymes	Quenching Mechanism	Detection Range	Reference
CdSe/ZnS@ SiO_2_	Not given	GOD	H_2_O_2_	0.5–3 mM	[30]
CdSe/ZnS	MSA	GOD	Acidic change	0.2–10 mM or 2–30 mM	[12]
CdTe	GSH	GOD	H_2_O_2_	0.05–1.0 mM	[31]
CdSe/ZnS	TGA	GOD/HRP	H_2_O_2_	0.045–10 mM	Our proposed method
